# Allogeneic stem cell transplantation in multiple myeloma: is there still a place?

**DOI:** 10.3389/fonc.2024.1402106

**Published:** 2024-06-04

**Authors:** Carmine Liberatore, Francesca Fioritoni, Mauro Di Ianni

**Affiliations:** ^1^ Hematology Unit, Department of Oncology and Hematology, Ospedale Santo Spirito, Pescara, Italy; ^2^ Department of Medicine and Sciences of Aging, University of Chieti-Pescara, Chieti, Italy

**Keywords:** allogeneic transplantation, multiple myeloma, immunotherapy, relapsed refractory multiple myeloma, alternative donor

## Abstract

The introduction of novel agents dramatically improved response and outcomes of multiple myeloma (MM) and led to a sharp decline in the use of allogeneic hematopoietic stem-cell transplantation (allo-HSCT). Thus, recent guidelines do not recommend anymore allo-HSCT as consolidation in the first-line treatment of newly diagnosed MM, even in high-risk patients. In a relapsed/refractory setting, allo-HSCT is not routinely recommended but should only be performed within clinical trials in young and high-risk patients. Nonetheless, allo-HSCT still represents a potential curative approach that has been used for decades in the treatment of MM and plasma cell neoplasms with favorable results and may still represent a treatment option for carefully selected patients. Despite that promising results were obtained with CAR T-cell therapies and bispecific antibodies in triple- and penta-exposed/refractory MM, these patients will inevitably relapse. To date, less is known about outcomes of allo-HSCT in patients exposed to novel immunotherapeutic drugs. Therefore, allo-HSCT could represent a reasonable treatment choice for younger and high-risk patients who have relapsed after CAR T-cell therapies and bispecific antibodies as well as an alternative for patients not eligible to these treatments and in those countries where immunotherapies are not yet available. In the choice of conditioning, reduced intensity conditioning regimens are currently recommended for the lower toxicity and mortality. Moreover, the use of alternative donors, particularly haploidentical, has progressively increased in last years with results comparable to full matched donors. Finally, post-transplantation maintenance strategies are encouraged whenever feasible.

## 1 Introduction

Allogeneic hematopoietic stem cell transplantation (allo-HSCT) still represents a curative treatment option for many hematological malignancies. Although its use has largely remained unchanged or increased in acute leukemia and myeloid neoplasms, recent years saw a sharp decline of allo-HSCT in multiple myeloma. (1–3) Since the introduction of novel agents such as proteasome inhibitors (PI), immunomodulating agents (IMIDs), monoclonal antibodies (MoAbs), and, ultimately, bispecific antibodies (BiAbs) and CAR-T cell therapies, response and outcomes of patients with MM have dramatically improved, thus leaving less and less room for allo-HSCT. (4) Nonetheless, allo-HSCT has been used for decades in the treatment of MM and plasma cell neoplasms with favorable results and may still represent a treatment option for individual patients.

## 2 Graft versus myeloma effect

The rationale for allo-HSCT in MM relies on both the cytotoxic effect of conditioning regimen and the long-term immunological control of the donor’s T lymphocytes against tumor-associated antigens (TAA). Although less intense than in other hematological malignancies, a Graft versus Tumor effect was also reported in MM. Actually, the donor’s CD8+ T cells proved able to recognize surface TAA on neoplastic plasma cells, thus granting significantly lower relapse incidence and longer survival. (5) Moreover, the occurrence of GvHD post allo-HSCT correlated in many trials with better outcome as an indirect evidence of Graft versus Myeloma effect (GvM). (6, 7) In the phase III BMT CNT 0102 trial, those patients with chronic GvHD had a significantly lower cumulative incidence of relapse. (8) Finally, in patients with MM persistent or relapsed after allo-HSCT, the administration of donor lymphocyte infusions (DLI) at escalating doses resulted in deeper response and longer survivals. (9–11) A multicenter retrospective study showed that among 61 patients with MM post allo-HSCT, the administration of prophylactic DLI deepened response rates up to minimal residual disease negativity in 26% of patients, with limited GvHD and mortality related to procedures (12).

## 3 Conditioning regimen

Conditioning regimens initially used in multiple myeloma were myeloablative (MAC). Although effective against MM, MAC regimens were burdened by severe toxicity, with 2-year TRM rates up to 50%. (13, 14) The improvement of supportive therapies and, above all, the introduction in recent years of reduced-intensity conditioning (RIC) regimens led to a significant reduction in TRM and made allo-HSCT possible for many more patients with MM. In a retrospective study on behalf of EBMT among patients with MM receiving RIC regimens mainly based on fludarabine, TRM at 100 days and 2 years were 10% and 26%, respectively. GvHD prophylaxis relied on either antithymocyte globulin (ATG) or alemtuzumab, the latter still determining a slightly higher TRM due to infectious mortality in the early post transplantation period. Nonetheless, in the whole cohort, 3-year progression-free survival (PFS) and overall survival (OS) were 21% and 41%, respectively. Notably, chronic GvHD was associated with longer OS and PFS, whereas chemoresistant and heavily pretreated patients benefited less from allo-HSCT. (7) A favorable TRM rate less than 20% after RIC allo-HSCT was reported in subsequent prospective trials. (15) Despite that the rationale of RIC allo-HSCT mainly relies on GvM, the cytotoxic effect of chemotherapy can be retained by the sequential approach of tandem autologous/allogeneic stem-cell transplantation as reported in several clinical trials. (16–20) Moreover, the alkylating agent treosulfan was included in RIC regimens for MM with the aim to further reduce toxicities. (21, 22) In a retrospective analysis comparing treosulfan-based RIC to “standard” RIC and MAC regimens in patients undergoing first allo-HSCT, treosulfan showed high engraftment rates and a favorable safety profile: non-relapse mortality (NRM) at 5 years was 17% overall and 9% when considering upfront allo-HSCT. Both treosulfan-based and “standard” RIC showed significantly higher OS when compared to MAC, although the benefit of treosulfan-based RIC was greater in the upfront setting. No differences emerged in terms of OS and PFS between conditioning regimens in patients with relapsed/refractory multiple myeloma (RRMM) (23).

Further attempts were made to improve efficacy of RIC regimens through the introduction of novel agents.

Proteasome inhibitors (PI) are key drugs in the treatment of MM. In addition to strong anti-myeloma effect, the first-in-class PI bortezomib showed immunomodulating properties and inhibitory effects on alloreactive T cells in murine models of allo-HSCT. (24) Notably, bortezomib showed a dual effect depending on the timing of administration. While an early administration together with conditioning chemotherapy resulted in increased disease control, the prolonged administration of bortezomib after allo-HSCT led to a harmful increase in acute GvHD, especially gastrointestinal and dependent on increased levels of TNFα and INFγ. (25) Two prospective clinical trials assessed bortezomib combined with a fludarabine and melphalan-based RIC in high-risk MM, followed by bortezomib maintenance starting at least after 50 days from allo-HSCT. At 1 year, the incidence of grade III–IV acute GvHD ranged between 10% and 25% and NRM approached 25%. OS and PFS at 2 years were 64% and 31%, respectively. Bortezomib both enhanced RIC efficacy and improved GvHD prophylaxis with limited toxicities. (26, 27) As long-term disease control was still suboptimal, a subsequent phase II trial tested maintenance with bortezomib and lenalidomide to decrease the risk of relapse. Despite that relapse rate was lower than previous studies (28.5% at 2 years), a limited number of patients actually proceeded to and completed maintenance due to the greater toxicity of lenalidomide post transplantation (28).

The radiosensitivity of neoplastic plasma cells favored the inclusion of radioimmunotherapy in RIC regimens. A phase I trial combined the anti-CD45 radio-labeled drug 90Y-DOTA-BC8 to fludarabine and low-dose total body irradiation (TBI) followed by allo-HSCT in patients with MM and high-risk features. Despite the relatively low expression of CD45 antigen on plasma cells, 90Y-DOTA-BC8 exerts a crossfire effect by targeting adjacent hematopoietic cells in bone marrow. Among 14 treated patients, the incorporation of 90Y-DOTA-BC8 to RIC was well tolerated, with manageable non-hematological toxicities and TRM of 0% at 100 days. Radioimmunotherapy also prolonged disease response. After a median follow-up of 5 years, PFS was 41% and OS was 71%. (29) Another strategy of indirect bone marrow irradiation is based on rhenium-188-labeled antibody targeting CD66 antigen (re-188-antiCD66) preferentially expressed on myeloid cells. There were 30 patients with heavily pretreated MM who received re-188-antiCD66 coupled with fludarabine-based RIC prior to allo-HSCT. At 2 years, PFS and OS were 43% and 55%, respectively, whereas 2-year NRM was 17%. Grade III–IV acute GvHD and severe chronic GvHD were reported in 26% and 17% of patients, respectively. In addition to expected mucositis and hematological toxicity, renal toxicity was the most frequent adverse event (7% grade III–IV acute renal failure and 10% grade III–IV chronic renal failure) due to dissociation of rhenium-188 from monoclonal antibody in kidneys (30).

## 4 Allogeneic HSCT in newly diagnosed multiple myeloma

Before the introduction of novel agents, allo-HSCT has been used for a long time as consolidation therapy after induction treatment in newly diagnosed multiple myeloma (NDMM). Several prospective trials compared allo-HSCT to ASCT in this setting. An Italian study enrolled 162 patients with NDMM to receive either RIC allo-HSCT or ASCT as consolidation treatment after a chemotherapy-based induction and first ASCT. Patients were assigned between two arms depending on the availability of an HLA-identical sibling. (16) Any difference emerged in terms of TRM between allo-HSCT and ASCT, but disease-related mortality was higher in tandem-ASCT cohort (43% vs 7%, p < 0.001). After a median follow-up of 7 years, both median OS and PFS were significantly longer among patients with HLA-identical sibling vs those without a suitable donor (not reached vs 4.25 years, p = 0.001; and 2.8 vs 2.4 years, p = 0.02; respectively) as well as among patients who actually received allo-HSCT vs those treated with tandem-ASCT (not reached vs 5.3 years, p = 0.02; and 39 vs 33 months, p = 0.02; respectively). Notably, 53% of patients in complete remission after allo-HSCT compared with 19% after tandem-ASCT maintained response at last follow-up. (17) Similarly, a PETHEMA study enrolled patients with NDMM and incomplete response after a first ASCT to receive either a second ASCT (n = 85) or a RIC allo-HSCT (n = 25), depending on the availability of an HLA–identical sibling donor. The study showed higher CR rates (40% vs 11%, p = 0.001) and a trend toward longer median PFS (not reached vs 31 months, p = 0.08) in the allo-HSCT cohort, whereas no difference emerged in TRM and OS. (31) In a prospective trial on behalf of EBMT, ASCT followed by RIC allo-HSCT (n = 108) was compared with single (n = 145) or tandem-ASCT (n = 104) in NDMM. After a median follow-up of 61 months, 5-year PFS and OS were significantly longer in the allo-HSCT cohort compared with patients treated with ASCT (35% vs 18%, p =0.001, and 65% vs 58%, p = 0.006, respectively), as was the incidence of disease relapse (49% vs 78%, p = 0.003). (18) As opposite, two different prospective trials failed to show a survival benefit in favor of allo-HSCT. After a median follow-up of 77 months, the HOVON-50 study showed comparable response rates and survivals between 138 patients without an HLA-identical sibling donor and 122 patients with a donor. A trend toward longer PFS emerged among the 99 patients who actually received allo-HSCT compared with 112 patients treated with single ASCT and thalidomide maintenance, whereas NRM at 6 years was 16% after allo-HSCT vs. 3% after ASCT (p < 0.001). (32) The prospective multicenter phase III BMT CNT 0102 trial also reported similar 3-year OS (77% vs. 80%) and PFS (43% vs. 46%) between patients diagnosed with standard-risk NDMM and treated with allo-HSCT vs. tandem-ASCT, in both intention-to-treat and as-treated population. (8) Results have been confirmed in a long-term analysis with over 10 years of follow-up. (33) A large retrospective Japanese registry study confirmed no survival advantage between the two transplantation approaches. (20) More recently, Kröger et al. reported results of a German multicenter phase II trial comparing allo-HSCT vs. tandem ASCT in NDMM on the basis of donor availability and evaluating the role of thalidomide maintenance and DLI post transplantation. (34) Among 217 enrolled patients, 65% received a bortezomib-based induction regimens. At 4 years, patients in the allo-HSCT cohort experienced higher NRM (13% vs. 2%; p = 0.044) but a lower relapse rate (40% vs. 63%; p = 0.04). Nonetheless, the lower incidence of disease relapse did not translate into longer PFS and OS at both 4 years (47% vs. 35%, p = 0.26, and 66% vs. 66%, p = 0.91, respectively) and 8 years (43% vs. 21%, p = 0.1, and 52% vs. 50%, p = 0.87, respectively). Prophylactic DLIs at escalating doses were given in 58 patients (50.4%) in the allo-HSCT cohort, but without improvement in PFS compared with those patients who did not receive DLI. Incidence of grade III–IV acute GvHD and chronic GvHD at 4 years was 6% in 61%, respectively. No increase in GvHD was observed following DLI and thalidomide maintenance. Although the relapse rate was lower in the allo-HSCT cohort and no relapse was observed after 5 years from transplantation, the trial failed to show improved survival with allo-HSCT. The deeper response rates observed after the bortezomib-based induction, the addition of thalidomide maintenance, and the greater proportion of patients who received allo-HSCT due to improved availability of matched unrelated donors (MUD) might have contributed to mitigating differences between arms and lowering the statistical power of the study. (34) Importantly, what emerges from these reported trials is that allo-HSCT is characterized by greater early TRM but lower risk of long-term disease relapses. Therefore, an appropriate follow-up of at least 5 years is needed to see the real benefit of allo-HSCT. For this purpose, a pooled analysis of 1,338 patients data provided an extended follow-up of the four major prospective trials. After a median follow-up of 118.5 months, NRM at 10 years was confirmed to be higher in the allo-HSCT group (19.7% vs. 8.3%, p < 0.001), but 10-year OS and PFS were significantly longer in patients who received allo-HSCT compared with ASCT (44.1% vs. 36.4%, p = 0.01; and 18.7% vs. 14.4%, p = 0.06). Moreover, survival of patients relapsed after allo-HSCT was significantly longer than after ASCT (62.3 months vs. 41.5 months, p <0.001), probably due to a better immunological fitness and enduring GvM effect in subsequent lines of treatment. (15) Results of prospective and retrospective studies on allo-HSCT in NDMM are summarized in **Table 1**.

**Table 1:** Prospective and retrospective studies on allogeneic HSCT in newly diagnosed multiple myeloma.

Study	Type of study	Pts (n)	Median age (years)	Induction regimen	Prior ASCT	Conditioning regimen	MRD MUD	ORR CR	TRM	Median follow up	OS	PFS	Acute GvHD	Chronic GvHD	Advantage of allo-HSCT over ASCT
Bruno (17)	Prosp	80	54	VAD	Yes	RIC 100%	100% 0%	86% 55%	16%	7 years	NR	33.6 months	43%	32%	Yes
Rosiñol (31)	Prosp	25	52	VBMCP/ VBAD	Yes	RIC 100%	100% 0%	- 40%	16%	5 years	NR	NR	32%	66%	Yes
Björkstrand (18)	Prosp	108	54	VAD	Yes	RIC 100%	100% 0%	93% 50%	16%	5 years	65% at 5 years	35% at 5 years	31%	54%	Yes
Lokhorst (32)	Prosp	122	54	VAD/ TAD	Yes	RIC 100%	100% 0%	- -	16%	6 years	55% at 6 years	28% at 6 years	48%	64%	No
Giralt (33)	Prosp	189	53	VAD/ T/B	Yes	RIC 100%	100% 0%	- 58%	20%	10 years	59% at 6 years	22% at 6 years	26%	54%	No
Kawamura (20)	Retro	89	49	–	Yes	MAC 12% RIC 88%	59% 24%	–	–	–	54% at 6 years	–	–	–	No
Kröger (34)	Prosp	132	51	B	Yes	RIC 100%	60% 40%	92% 71%	13%	10 years	52% at 8 years	43% at 6 years	38%	61%	No
Costa (15)	Retro	439	53	–	Yes	RIC 100%	100% 0%	–	19.7%	10 years	44% at 10 years	18% at 10 years	–	–	Yes

ASCT, autologous stem-cell transplantation; MRD, matched related donor; MUD, matched unrelated donor; ORR, overall response rate; CR, complete remission; OS, overall survival; PFS, progression-free survival; GvHD, graft-versus-host disease; allo-HSCT, allogeneic stem-cell transplantation; VAD, vincristine, adriamycin, dexamethasone; T, thalidomide-based induction; B, bortezomib-based induction; RIC, reduced intensity conditioning regimen; MAC, myeloablative conditioning regimen; NR, not reached; VBMCP, vincristine, carmustine, melphalan, cyclophosphamide, prednisone; VBAD, vincristine, carmustine, adriamycin, dexamethasone (VBAD).

Patients with high-risk NDMM by either chromosomal abnormalities or clinical characteristics have dismal outcomes with standard treatment. Therefore, in this setting, allo-HSCT has been employed in the attempt to improve survival. Initial retrospective studies reported similar survival rates in high-risk NDMM who underwent allo-HSCT compared with standard-risk patients treated with conventional treatments. (35–37) A meta-analysis including 8,698 patients from 61 clinical trials showed similar OS and PFS between high-risk patients who received allo-HSCT and patients with standard-risk disease treated with ASCT, suggesting that GvM might in part overcome the poor prognostic features. (38) Few prospective trials selectively explored the role of allo-HSCT in high-risk NDMM. The Intergroupe Francophone du Myelome (IFM) evaluated in two parallel prospective studies the allogeneic transplantation approach (IFM99–03) versus consolidation with tandem ASCT (IFM99–04) after a chemotherapy-based induction in high-risk NDMM defined as elevated beta2-microglobulin and chromosome 13 deletion. At a median of follow-up of 24 months, both median OS and EFS were comparable (35 months vs. 41 months, p = 0.27, and 25 months vs. 30 months, p = 0.56, respectively) between 65 patients in the IFM99–03 trial and 219 patients in the IFM99–04 trial. A trend for longer OS emerged in tandem ASCT cohort (47.2 vs. 35 months; p = 0.07). (39) An extended follow-up analysis did not confirm the superiority of RIC allo-HSCT compared with tandem ASCT, in part explained by the conditioning regimen before allo-HSCT based on busulfan, fludarabine, and high-dose ATG that might have reduced the GvM. (40) Similarly, the BMT CNT 0102 trial and the HOVON-50 trial failed to show an advantage in terms of OS and PFS of allo-HSCT over tandem ASCT consolidation in the high-risk population. (32, 33) Remission status at transplant also appeared to significantly correlate with long-term survival in high-risk NDMM undergoing allogeneic transplantation. (41, 42) Recently, a retrospective registry analysis included NDMM with del(17p) and/or t(4;14) undergoing either single ASCT (n = 446), tandem ASCT (n = 105), or ASCT/RIC allo-HSCT (n = 72). Donors were MRD in 54% and MUD in 46% of cases. Notably, the majority of patients (n = 431, 69.2%) already received an induction regimen containing bortezomib. The OS at 5 years for single ASCT, tandem ASCT, and ASCT/RIC allo-HSCT were 51%, 60%, and 67%, respectively (p = 0.187). Similarly, 5-year PFS were 17%, 33%, and 34%, respectively (p = 0.048), and 5-year NRM were 1%, 4%, and 10%, respectively. In multivariate analysis, in patients harboring t(4;14), both tandem ASCT and ASCT/RIC allo-HSCT granted longer PFS compared with single ASCT, whereas only tandem ASCT was associated with longer OS. Conversely, the poor prognostic impact of del(17p) was partly mitigated by ASCT/RIC allo-HSCT in terms of PFS (HR, 0.65; p = 0.097), but no significant difference in OS emerged between groups (43).

Major limits of the reported experiences on allo-HSCT in NDMM are the heterogeneity of disease and patients’ characteristics, the variability in the identification of high-risk features and staging systems, the diversity of induction regimens given before allo-HSCT, the short follow-up, and, above all, the lack of novel agents that currently represent the milestone in the treatment of NDMM. Therefore, upfront RIC allo-HSCT is not recommended in first-line treatment of standard-risk and high-risk NDMM. It may be considered in patients with very high-risk MM, only in the context of clinical trials (44–46).

## 5 Allogeneic HSCT in relapsed/refractory multiple myeloma

In patients with RRMM after first-line treatment, allo-HSCT has been use as a salvage treatment. To date, large prospective trials are lacking in this setting and the majority of evidence is based on retrospective studies. A report from EBMT actually showed between 1990 and 2012 a steady increase in the use of allo-HSCT for treatment of RRMM parallel to a progressive decline of upfront allo-HSCT. Among 3,405 patients who underwent salvage allo-HSCT, 5-year OS was 32%. (47) In the attempt to identify prognostic factors, another EMBT registry study analyzed outcomes of 413 patients with RRMM who received RIC allo-HSCT from MRD or MUD. Overall, 44.6% of patients had received at least two prior ASCT. Median OS and PFS were 24.7 and 9.6 months, respectively, whereas 1-year NRM was 21.5%. In multivariate analysis, CMV negative status in both donor and recipients and less than two prior ASCT significantly correlated with better outcomes. (48) In patients exposed to PI (bortezomib 100%) and IMIDs (lenalidomide 61.5%) and after a median of three prior lines of therapy, a Japanese study reported 3-year PFS and OS of 18.8% and 47.2%, respectively, whereas 3-year NRM was 23.4%. In multivariate analysis, older age (≥50 years) and incomplete response before allo-HSCT independently predicted worse PFS and OS. Although allo-HSCT appeared as a reasonable treatment option in heavily pretreated patients with RRMM, better results are obtained mainly in young patients with chemo-sensitive disease and in good response before transplantation. (49) Similarly, in a cohort of heavily pretreated patients with high-risk RRMM (57% have at least one abnormality among t(4:14), t(14:16), del17p or gain1q), salvage allo-HSCT showed 5-year OS and PFS of 66% and 48%, respectively. TRM at 5 years was low (9%), whereas incidence of grade II–IV acute and chronic GvHD accounted for 21% and 58%, respectively. Notably, in this cohort, the development of chronic GvHD predicted longer survival. (50) Conversely, the efficacy of allo-HSCT is limited in patients who have active disease at transplantation (median PFS and OS of 6 months and 23 months, respectively) as well as in those patients with early relapse (<12 months) after first-line treatment. (51–53) Compared with non-transplantation approaches, an Italian retrospective study showed a potential benefit for allo-HSCT in RRMM. Patients with MM in first relapse after ASCT received either allo-HSCT (n = 72) or salvage treatment with PI and IMIDs (n = 90) depending on donor availability. After a median follow-up of 110 months, PFS and OS at 7 years were significantly longer in the allo-HSCT group compared with the non-transplantation group (18% vs. 0%, p < 0.0001; and 31% vs. 9%, p < 0.0001; respectively). (54, 55) Interestingly, the benefit of allo-HSCT seems to persist in subsequent lines of treatments, possibly due to the lower immunological exhaustion of the donor’s immune system granting better response to further therapies. Indeed, a registry analysis by CIBMT reported longer post-relapse survival (44% vs. 35% at 6 years, p = 0.05) in patients with RRMM treated with ASCT/RIC allo-HSCT compared with tandem ASCT. (56) Although uncommon, a second allo-HSCT was also attempted in past years. The EBMT Chronic Malignancies Working Party retrospectively analyzed data of 215 patients who underwent a second allo-HSCT either for relapse (n = 159) or for graft failure (n = 56). In the relapse group, OS at 2 years and 5 years were 38% and 25%, respectively. The majority of patients (83%) received the second allo-HSCT from the same MRD. Despite a higher incidence of grade II–IV acute GvHD in those patients who received second transplantation from the same donor (50% vs. 22%, p = 0.03), the use of the same MRD conferred better outcomes in multivariate analysis (5-year OS 35% vs. 9%; p < 0.001). The interval between transplantations also influenced outcomes. Patients who received second allo-HSCT within 2 years from the first procedure had shorter survival compared with late relapses. (57) Results of retrospective studies on allo-HSCT in RRMM are summarized in **Table 2**.

**Table 2:** Retrospective studies on allogeneic HSCT in relapsed/refractory multiple myeloma.

Study	Type of study	Pts (n)	Median age (years)	Median LOT (n)	Prior ASCT	Conditioning regimen	MRD MUD	ORR CR	TRM	Median follow-up	OS	PFS	Acute GvHD	Chronic GvHD
Sobh (47)	Retro	3,405	54	–	Yes	MAC 25% RIC 75%	46% 14%	–	29%	3 years	33% at 5 years	15% at 5 years	–	–
Auner (48)	Retro	413	54	–	Yes	–	58% 39%	–	28%	–	24.7 months	9.6 months	49%	47%
Kawamura (49)	Retro	65	49	3	Yes	MAC 26% RIC 74%	40% 60%	51% 27%	23%	–	47% at 3 years	18% at 3 years	45%	44%
Jurgensen-Rauch (50)	Retro	37	52	2	Yes	RIC 100%	59% 41%	–	9%	4.6 years	66% at 5 years	48% at 5 years	21%	58%
Patriarca (55)	Retro	72	53	–	Yes	RIC 100%	35% 65%	–	27%	9 years	31% at 7 years	18% at 7 years	33%	65%
Strassl (58)	Retro	38	55	7	Yes	MAC 55% RIC 45%	26% 34%	89% 68%	16%	3 years	51.4 months	13.6 months	42%	12%

ASCT, autologous stem-cell transplantation; MRD, matched related donor; MUD, matched unrelated donor; ORR, overall response rate; CR, complete remission; OS, overall survival; PFS, progression-free survival; GvHD, graft-versus-host disease; allo-HSCT, allogeneic stem-cell transplantation; RIC, reduced intensity conditioning regimen; MAC, myeloablative conditioning regimen.

Similarly to the first-line setting, the introduction of novel agents in recent years has significantly improved the prognosis of RRMM; therefore, the indication for allo-HSCT is significantly reduced in this setting. Allo-HSCT might represent a possible treatment option in carefully selected RRMM who have exhausted other therapeutic alternatives, especially in case of chemo-sensitive disease and late relapses from previous ASCT. Then, allo-HSCT is not routinely recommended for RRMM and it should possibly be performed in the context of a clinical trial (44–46, 59).

## 6 Alternative donors

The studies reported so far in the treatment of both NDMM and RRMM have mainly included allo-HSCT from MRD and, to a lesser extent, MUD. However, approximately one-third of candidates to allo-HSCT do not have a full matched donor. Therefore, over the years there was a progressive increase in the use of alternative donors such as mismatched unrelated donors (MMUD), haploidentical donors (haplo), and cord blood units (CBU). (2, 3) A German multicenter prospective study reported on 49 patients with RRMM after prior ASCT and treated with RIC regimen based on fludarabine and melphalan followed by allo-HSCT from either MUD or MMUD. Overall, OS and PFS at 4 years were 26% and 20%, respectively, and were significantly longer for patients with persistent CR at day +100 (41% vs. 7%, p = 0.04; and 56% vs. 16%, p = 0.02). The incidence rates of acute and chronic GvHD were 25% and 25%, respectively, and compared favorably with those previously reported with MRD. However, 1-year TRM was significantly higher for MMUD than MUD (53% vs. 10%, p = 0.001). (60) A subsequent retrospective study confirmed favorable long-term outcomes but with limited TRM rates of 12%, similar between donor’s types. (61) Then, a large retrospective study by EBMT included 570 patients with MM early relapsed after single or tandem ASCT. Patients received RIC allo-HSCT from MUD (n = 419), MMUD (n = 93), and CBU (n = 58). No significant difference emerged in terms of OS, PFS, and TRM according to type of donors (5-year OS was 33%, 39%, and 25%, respectively; 5-year PFS was 14%, 27%, and 4%, respectively; TRM was 22%, 33%, and 27%, respectively). Notably, a trend for better long-term survivals emerged within the MMUD cohort compared with MUD possibly related to a stronger GvM, whereas allo-HSCT from CBU was burdened by greater toxicities (62).

The cord blood unit has the advantage of being a readily available graft source but is burdened by technical difficulties in transplantation and greater complications compared with allo-HSCT from other donors that limit its wide application. Two registry analyses on behalf of the Eurocord and the Japan Society for Hematopoietic Cell Transplantation including over 180 patients with RRMM allografted with CBU showed high TRM ranging 29%–39% but favorable rates of PFS and OS ranging 14%–25% and 31%–40% at 3 years, respectively (63, 64).

Haploidentical donors are the source of alternative donors with the greatest increase in use for allo-HSCT in the last decades, considering the wide availability of familiar donors as well as the relative ease of execution. In the setting of multiple myeloma, a collaborative retrospective study from EBMT and CIBMTR identified 96 patients with RRMM treated with allo-HSCT from haplo. Among pretreated patients mainly relapsed post ASCT, NRM was 21% at 1 years, whereas 2-year OS and PFS were 48% and 17%, respectively. Following a GvHD prophylaxis based on post-transplantation cyclophosphamide (PTCy), the cumulative incidence of grade II–IV acute GvHD was 39% at 100 days, whereas chronic GvHD occurred in 46% of patients. (65) Similar results were also reported by a smaller retrospective Italian study. (66) Notably, the GvHD prophylaxis based on PTCy was initially developed for haploidentical allo-HSCT but has been subsequently applied also to other graft sources. Among 295 patients with RRMM undergoing allo-HSCT from different grafts (MRD, n = 67; MUD, n = 72; MMUD, n = 27; haplo, n = 129), PTCy was used as GvHD prophylaxis in combination with calcineurin inhibitors (n = 239, 81%) and/or mycophenolate mofetil (n = 184, 77%). In this different setting of patients, incidence of grade II–IV acute GvHD was 30% at day +100 whereas chronic GvHD occurred in 27% of cases. Notably, no differences were observed according to donor type neither in GvHD incidence nor in outcomes. In multivariate analysis, the use of MRD conferred better OS compared with haplo, whereas only a trend was reported for MUD. Therefore, PTCy appeared as a wide applicable platform for GvHD prevention granting for favorable outcomes even in non-haplo settings (67).

## 7 The role of maintenance

Proteasome inhibitors such as bortezomib have been widely given as maintenance treatment after ASCT in MM. Considering both anti-myeloma and immunologic properties, bortezomib has been used after allo-HSCT in the attempt to maintain response and prolong survival. (68–70) In a prospective phase II trial, 39 patients with high-risk NDMM received a bortezomib-based induction followed by tandem ASCT/RIC allo-HSCT and then maintenance with bortezomib. Treatment was well tolerated (NRM 12%), with limited toxicities and incidence of grade II–IV acute GvHD and chronic GvHD of 26% and 57%, respectively. The 5-year PFS was 41% and 5-year OS was 80%. In a multivariate analysis, minimal residual disease positivity both prior to allo-HSCT (p = 0.037) and after 3 months from transplantation (p = 0.001) strongly predicted disease relapse. (71) A recent retrospective study by the same group selectively analyzed the role of bortezomib in reducing the incidence and severity of GvHD post allogeneic transplantation in a cohort of 46 NDMM patients compared with 61 patients who did not received maintenance. According to NIH 2014 criteria, patients in the bortezomib group had lower incidence of both overall and moderate/severe chronic GvHD than the control group (61.2% vs. 83.6%, p = 0.001; 44.5% vs. 77.0%, p = 0.001, respectively). Moreover, bortezomib favored a lower use of systemic steroids (45.1% vs. 76.4%, p < 0.001) and allowed a greater number of patients to discontinue immunosuppression (77% vs. 56%, p = 0.046). (72) As in the autologous transplantation setting, maintenance with the second-generation PI ixazomib has been tested in high-risk NDMM after allo-HSCT. Although incidence rates of acute and chronic GvHD were limited, a phase II double-blind trial did not show a survival advantage compared with the placebo group. (73) Thus, further studies are needed to explore the effective role of ixazomib in the allogeneic setting.

The other cornerstone in the treatment of MM is represented by IMIDs. The first-in-class thalidomide proved feasible and effective as maintenance post allo-HSCT as previously reported. (34) Lenalidomide is currently approved for post ASCT maintenance. (74) In the post allogeneic transplantation setting, lenalidomide promotes immune-mediated GvM by enhancement of NK cell activation as well as induction of a strong anti-myeloma activity, as well as an increase in the release of IFN-γ by CD4+ and CD8+ T cells and late expansion of regulatory T cells. (75) The prospective HOVON-76 study reported promising results (2-year OS 93%, 2-year PFS 60%) in high-risk NDMM receiving lenalidomide 10 mg on days 1 to 21 of a 28-day cycle after allo-HSCT, but maintenance was burdened by relevant acute and chronic GvHD incidence (53%) and toxicities that led to premature discontinuation of lenalidomide in 47% of participants. (76) Similar results were reported by other prospective studies. (77–79) Then, lowering doses of lenalidomide to 5 mg daily showed better tolerability without negatively impacting survival outcomes (PFS and OS 61% and 79% at 2 years, respectively) (75).

The combination of novel agents such as PI or IMIDs together with DLI has been explored as post-transplantation immunotherapy in those patients failing to achieve CR after allo-HSCT. Among 32 enrolled patients, approximately 60% achieved CR with acceptable rates of both grade II–IV acute and chronic GvHD (33% and 17%, respectively). Notably, deepening the response up to CR significantly predicted for longer 5-year PFS and OS (53% vs. 35%, p = 0.03; and 90% vs. 62%; p = 0.06, respectively), thus highlighting the importance of achieving and maintaining deeper response even in the post allogeneic transplantation setting (11).

## 8 Allogeneic HSCT in the era of novel drugs

The introduction in the last decade of novel agents such as PI, IMIDs, and anti-CD38 MoAbs alone or in combination has dramatically improved outcomes for both NDMM and RRMM. (4) Nonetheless, multiple myeloma eventually relapses. Currently, these patients represent an unmet clinical need with dismal outcome: median OS is approximately 12 months in triple-exposed/refractory patients but reaches less than 6 months in those penta-exposed/refractory. (80, 81) Unfortunately, data on allo-HSCT in this setting are very scarce. A recent single-center retrospective experience by Strassl et al. reported on 38 patients with heavily pretreated RRMM who consecutively received allo-HSCT between 2013 and 2022. The median number of previous lines of therapy was 7 (range, 4–13); 74% was triple-class exposed, whereas 24% was triple-class refractory. The conditioning regimen was MAC in 55% of patients whereas 45% of them received RIC mainly based on TBI. The source of donor was heterogenous: MRD 26%, MUD 34%, MMUD 8%, and haplo 34%. The overall response rates (at least PR) to bridging therapy were 87% and 23% achieved CR/sCR. Notably, allo-HSCT was able to deepen response only in those patients who initially responded to bridging therapy, whereas the proportion of refractory patients remained almost unchanged before and after allo-HSCT (13% vs. 11%). After transplantation, only 26% of patients could receive maintenance considering previous drug exposure and expected toxicities whereas 37% of them received DLI. Overall, NRM was 16% and allo-HSCT appeared as a feasible choice in advanced-stage MM. After a median follow-up of 37.5 months for survivals, median PFS and OS were 13.6 months and 51.4 months, respectively. In multivariate analysis, remission status before allo-HSCT (VGPR or better) as well as the absence of high-risk cytogenetic abnormalities by FISH significantly predicted longer survivals. Nonetheless, 58% of patients relapsed after allo-HSCT and the majority of them could receive at least one further treatment. As expected, prognosis was dismal, with an estimated OS of 22.6 months. Among 41 different treatment regimens, five patients received belantamab mafodotin (23%), seven patients were treated with teclistamab (32%), and one patient subsequently also received talquetamab. Notably, all these novel agents proved feasible when used after allo-HSCT, without unexpected severe toxicity or GvHD flares (58).

Novel therapies such as CAR-T and BiAbs have shown promising results in triple- and penta-exposed/refractory MM. (82–85) However, despite high response rates, patients still eventually relapse and few data are available on salvage treatments in this setting. In a recent retrospective analysis, Van Oekelen et al. reported on 79 patients with multiple myeloma relapsed following treatment with BCMA-directed CAR T. After a median follow-up of 21.3 months, median OS from relapse was 17.9 months. In multivariate analysis, penta-drug refractoriness was associated with worse outcomes (median OS 13.9 vs. 29.9 months, p = 0.018), whereas achievement of at least a partial response to salvage regimen predicted longer OS compared with non-responding patients (29.9 vs. 14.6 months, p = 0.028). In absence of a standard of care, patients received more than 200 different salvage regimens. The most common were CAR T and BiAbs (35 patients, 44.3%) both BCMA-directed and non–BCMA- directed, with overall response rates (ORR) of 91.4% and median OS not reached at data cutoff. Notably, seven patients received allo-HSCT as salvage therapy, obtaining high response rates (ORR 100%) and favorable outcomes (median OS 23.2 months). (86) Similar results (ORR 42%, median OS 18 months) were reported in another single-center analysis of 68 patients relapsed after commercially available CAR-T. (87) Thus, although additional T-cell–engaging therapies showed clinical activity in case of MM relapsed after CAR T, allo-HSCT also appears as a reasonable treatment option in this setting and, moreover, in all that countries where CAR T and BiAbs are not yet available and for those patients who are not eligible to receive these treatments.

## 9 Conclusion

The use of allogeneic transplantation in multiple myeloma has dramatically dropped in recent years following the introduction of novel agents. Thus, the role of allo-HSCT as consolidation in the first-line treatment of NDMM has disappeared, even in high-risk patients. In RRMM, allogeneic transplantation is not routinely recommended and should only be performed in carefully selected high-risk patients within clinical trials. Less is known about allo-HSCT in patients exposed to new drugs which currently represent the majority of relapsed patients. Therefore, specific studies are encouraged in triple- and penta-exposed/refractory population. Although promising results are reported with CAR T and BiAbs in this setting, patients still eventually relapse. Then, allogeneic transplantation could represent a reasonable treatment choice for younger and high-risk patients who have relapsed after CAR T and BiAbs as well as for those patients not eligible to CAR T and BiAbs and in those countries where these treatments are not yet available. In the choice of conditioning, RIC regimens are widely recommended for the lower TRM. The use of alternative donors, particularly haploidentical, has demonstrated favorable results compared with full matched donors. Finally, post-transplantation maintenance strategies are encouraged whenever feasible.

## Author contributions

CL: Conceptualization, Writing – original draft, Writing – review & editing. FF: Conceptualization, Writing – original draft, Writing – review & editing. MI: Conceptualization, Writing – original draft, Writing – review & editing.
